# Regulatory effect of N6-methyladenosine on tumor angiogenesis

**DOI:** 10.3389/fimmu.2024.1453774

**Published:** 2024-09-04

**Authors:** YuYan, Enwu Yuan

**Affiliations:** Department of Laboratory Medicine, The Third Affiliated Hospital of Zhengzhou University, Zhengzhou, Henan, China

**Keywords:** N6-methyladenosine, tumor, noncoding RNA, angiogenesis, regulatory role, anti-tumor therapy

## Abstract

Previous studies have demonstrated that genetic alterations governing epigenetic processes frequently drive tumor development and that modifications in RNA may contribute to these alterations. In the 1970s, researchers discovered that N6-methyladenosine (m^6^A) is the most prevalent form of RNA modification in advanced eukaryotic messenger RNA (mRNA) and noncoding RNA (ncRNA). This modification is involved in nearly all stages of the RNA life cycle. M^6^A modification is regulated by enzymes known as m^6^A methyltransferases (writers) and demethylases (erasers). Numerous studies have indicated that m^6^A modification can impact cancer progression by regulating cancer-related biological functions. Tumor angiogenesis, an important and unregulated process, plays a pivotal role in tumor initiation, growth, and metastasis. The interaction between m^6^A and ncRNAs is widely recognized as a significant factor in proliferation and angiogenesis. Therefore, this article provides a comprehensive review of the regulatory mechanisms underlying m^6^A RNA modifications and ncRNAs in tumor angiogenesis, as well as the latest advancements in molecular targeted therapy. The aim of this study is to offer novel insights for clinical tumor therapy.

## 1 Introduction

Tumor angiogenesis is an uncontrolled and persistent process that plays a crucial role in tumor growth and metastasis (1). Unlike normal angiogenesis, which is regulated through complex biological processes, tumor angiogenesis has abnormal characteristics, such as irregular morphological structures, disorganized arrangements of endothelial cells, and unstable vascular walls. These abnormalities result in a hypoxic state and the accumulation of metabolic waste within the tumor while also providing nutrients and pathways for tumor cell proliferation and metastasis (2). The regulation of tumor angiogenesis involves multiple signaling pathways and molecular mechanisms (3). Recent studies have highlighted the significant regulatory roles of m^6^A modifications and noncoding RNAs (ncRNAs) in this process.

In recent years, an increasing number of studies have shown that methylations play crucial roles in the regulation of tumor angiogenesis (4, 5). Methylation is a process that involves the addition of methyl groups to DNA or RNA molecules. This modification can affect gene expression by altering chromatin structure and binding sites for transcription regulatory factors. In tumors, the patterns of methylations on DNA and RNA often undergo changes, which are closely associated with tumor progression, invasiveness, and patient prognosis (6–8).

**Figure 1:**
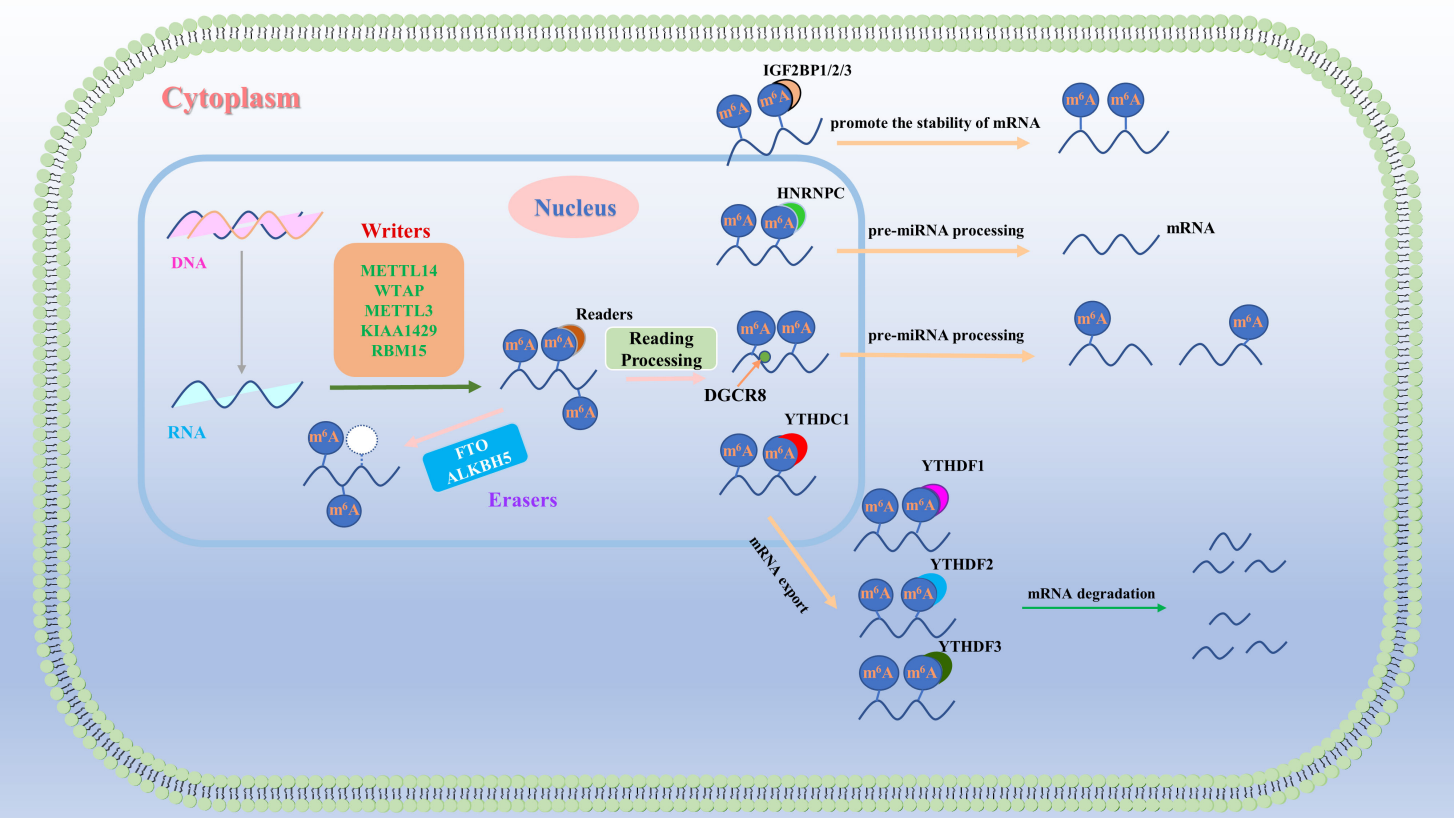
Regulatory mechanism of m^6^A RNA methylation. M^6^A RNA methylation is a dynamic process that is governed primarily by three groups of proteins: “writers,” “erasers,” and “readers.” “Writers” and “erasers” are located predominantly in the nucleus, whereas some “readers,” such as HNRNPs and YTHDC1, also function in the nucleus. Other “readers,” including YTHDC2 and IGF2BP1/2/3, are found in both the cytoplasm and the nucleus, whereas “readers”, such as YTHDF1/2/3, are exclusively present in the cytoplasm. These three groups of proteins collaboratively regulate the output, stability, translation, and degradation of RNA.

M^6^A is a prevalent chemical modification observed in RNA molecules (9). M^6^A modification involves the addition of a methyl group to the adenine base of RNA molecules. This modification is widely distributed among eukaryotes, including humans, mice, and fruit flies. M^6^A modification is a dynamic process in which methyl groups are added to RNA molecules by methyltransferases and removed by demethylases (10). This modification can impact various cellular processes, such as RNA stability (11–13), posttranscriptional regulation (14, 15), translation (12, 16), and splicing (17–19) (**Figure 1**). Importantly, m^6^A modification can directly or indirectly influence the expression and function of factors related to tumor angiogenesis by regulating the transcription levels, RNA stability, and translation efficiency of key genes. For example, researchers have discovered that m^6^A modification can enhance or inhibit the regulatory effects of specific ncRNAs on crucial biological processes such as endothelial cell proliferation, migration, and lumen formation. Currently, m^6^A is increasingly recognized as a promising biomarker for cancer detection and prevention because of its potential clinical value in cancer research.

NcRNAs encompass a group of RNA molecules that do not possess protein-coding capabilities but instead serve as crucial regulators within cells (20). Emerging research has highlighted the significant role of ncRNAs in the regulation of tumor angiogenesis. Specifically, certain ncRNAs interact with the mRNAs of genes associated with angiogenesis, thereby modulating their stability and posttranscriptional modification levels. These interactions ultimately impact the process of tumor angiogenesis.

While studies have examined the regulatory roles of m^6^A modifications and ncRNAs in tumor angiogenesis, our current knowledge in this area remains limited. Therefore, further investigations into the mechanisms underlying the interaction between m^6^A modification and ncRNAs in the regulation of tumor angiogenesis are crucial. Therefore, in this review, we assess the regulatory effect of m^6^A on tumor angiogenesis and provide a theoretical basis for the development of novel targeted therapeutic strategies.

## 2 M^6^A, NcRNA and tumor angiogenesis

### 2.1 M^6^A

M^6^A is composed of a ribose with a purine base and a methyl group attached to the sixth nitrogen atom. It was not until 2012 that the genome-wide distribution of m^6^A was elucidated, and m^6^A is one of the most abundant modifications in eukaryotic mRNAs (11, 21, 22). M^6^A is considered the most common, frequent, and conserved internal modification, with an average of 1–2 m^6^A residues per 1000 nucleotides (23). M^6^A primarily occurs within the conserved motif RRACH (R = G or A and H = A, C, or U), which tends to be found in stop codons and 3′ untranslated regions (3′ UTRs) (24–27). The basic process of m^6^A modification involves the installation of methyl groups by “writer” methyltransferases, removal by demethylases known as “erasers,” and recognition by m^6^A-binding proteins called “readers,” thereby regulating RNA metabolism (28–31). This is a dynamic process (**Figure 1**). The m^6^A methyltransferase complex (MTC) consists of methyltransferase-like 3 (METTL3) (32, 33), METTL14 (34), the METTL3 adaptor protein wilms tumor 1-associated protein (WTAP) (35), and other associated proteins, including METTL7A/B (36), METTL5 (37, 38), the METTL5-tRNA MTase subunit 11–2 (TRMT112) complex (39, 40), Vir-like m^6^A methyltransferase associated protein (VIRMA/KIAA1429) (41, 42), RNA-binding motif protein 15 (RBM15) (43, 44), METTL16 (45), zinc finger CCCH domain-containing protein13 (ZC3H13) (46–48), CBLL1 (Cbl proto-oncogene like 1) (49), and zinc finger CCHC domain-containing protein 4 (ZCCHC4) (50). METTL3 is a key protein in this complex and was first identified as an S-adenosylmethionine-binding protein with methyltransferase activity (33). As a pseudomethyltransferase, METTL14 plays a crucial role in facilitating complex formation and RNA binding. *In vitro* and *in vivo* experiments have demonstrated that METTL3 and METTL14 form a heterodimer, and together, they catalyze m^6^A methylation, while their individual components exhibit lower activity (51). WTAP does not possess catalytic activity but can interact with METTL3 and METTL14, thereby regulating m^6^A levels during RNA transcription (34). Recently, METTL16 was shown to regulate splicing by targeting pre-mRNAs and various ncRNAs (52). VIRMA is capable of binding m^6^A with 3’UTRs, whereas ZC3H13 functions to induce the translocation of the writer complex into the nucleus. Recent reports have identified VIRMA and ZC3H13 as new components of the m^6^A methyltransferase complex that regulate the selectivity of m^6^A site modifications on mRNAs. These findings provide new insights into the regulatory mechanisms of m^6^A occurrence (53, 54). Fat mass and obesity-associated protein (FTO) was the first discovered m^6^A RNA demethylase (55), capable of interacting with various RNA molecules, including mRNA, snRNA, and tRNA. *In vitro* studies have shown that FTO effectively oxidizes and demethylates m^6^A and N6,2’-O-dimethyladenosine (m^6^Am) on RNA (56–58). However, despite its significant demethylation activity on m^6^A *in vitro*, its regulatory role on m^6^A under physiological conditions appears to be relatively limited. Increasing evidence suggests that FTO primarily regulates RNA function through the demethylation of m^6^Am (59). M^6^Am is typically situated near the 5’ cap structure of mRNA (60), and its demethylation is more prominent in the cytoplasm than in the nucleus, likely due to the differential subcellular localization of FTO. M^6^Am plays a critical role in regulating mRNA stability and translation efficiency (61–64). Studies indicate that FTO predominantly acts on m^6^Am within snRNA in the nucleus (65), while in the cytoplasm, it preferentially demethylates m^6^A at the 5’ end and within internal regions of mRNA (66). Notably, there is ongoing debate regarding whether FTO’s role in m^6^A demethylation involves reversible and dynamic regulation, underscoring the need for further in-depth research (59, 67). However, alkB homolog 5 (ALKBH5) exhibits comparable demethylation activity to m^6^A and shows a preference for consensus sequences (RRACH) that are consistent with m^6^A (43). Silencing or overexpression of these factors leads to an increase or decrease in m^6^A on mRNA, respectively. Knockout of FTO or ALKBH5 results in an overall increase in m^6^A levels in human cells. M^6^A readers interpret information on RNA methylations and participate in downstream processes such as translation and degradation of RNA. Different readers have distinct physiological functions. The identified readers can be classified into three categories: the first category includes readers with YTH domains (68, 69); the second category consists of HNRNPs (70), which possess the same RNA-binding domains (RBDs); and the third category of readers includes those with KH domains, RNA recognition motifs (RRMs), and arginine/glycine-rich (RGG) domains (71–73). These readers can all bind to m^6^A-modified mRNAs, including FMR1 and IGF2BP1-3. Interestingly, YTHDF1, YTHDF2, and YTHDF3 are the principal members of the YTHDF protein family (74, 75). Initial studies suggested that these proteins specifically bind to m^6^A sites, with YTHDF1 enhancing translation, YTHDF2 promoting mRNA degradation, and YTHDF3 both enhancing translation and facilitating mRNA degradation (76, 77). Additionally, YTHDF3 may have a potential role in regulating RNA transport, particularly in neurons or related systems (78). However, recent research has revealed that YTHDF1, YTHDF2, and YTHDF3 exhibit functional redundancy in regulating mRNA degradation (79). The YTHDF protein family collaboratively binds to m^6^A-modified sites to mediate this process, with all three proteins primarily promoting the degradation of m^6^A-modified mRNAs rather than directly enhancing translation (80). This finding prompts a reassessment of the roles of YTHDF proteins in RNA metabolism and may challenge our current understanding of m^6^A regulation (79, 81). The combined actions of writers, erasers, and readers form the m^6^A modification system that regulates the biological functions and metabolism of RNA (30, 63–65).

### 2.2 NcRNA

NcRNAs are pivotal regulators of gene expression that function independently of protein translation. These RNAs include small nuclear RNAs (snRNAs), small interfering RNAs (siRNAs), microRNAs (miRNAs), long noncoding RNAs (lncRNAs), and circular RNAs (circRNAs) (82, 83). These ncRNAs exert their regulatory effects through diverse mechanisms, such as posttranscriptional regulation, transcriptional repression, and chromatin remodeling (84). MiRNAs, which are typically 20–24 nucleotides in length, bind to the 3’ untranslated regions of target mRNAs, leading to mRNA degradation or translational inhibition, and are implicated in the pathogenesis of diseases, including cancer and cardiovascular disorders (85, 86). LncRNAs, which are typically longer than 200 nucleotides, regulate gene expression through multiple mechanisms. At the genomic level, lncRNAs modulate gene expression via classical pathways such as transcription, posttranscriptional mRNA processing, turnover, and translation. At the epigenomic level, lncRNAs further fine-tune genomic control by altering chromatin architecture and modifying the chemical properties of DNA and RNA. Additionally, lncRNAs transcend genomic and epigenomic regulation, adding an extra dimension of control by influencing both transcriptional and posttranscriptional processes (87). As a result, lncRNAs play pivotal roles in cellular development, tissue-specific gene expression, and the progression of various diseases (87, 88). CircRNAs, distinguished by their covalently closed loop structures, exhibit high stability and act as miRNA sponges, thereby modulating the activity of miRNAs and potentially regulating transcription factors (89, 90). Dysregulation of these ncRNAs is associated with a range of biological processes and diseases, highlighting their critical roles in gene regulatory networks and their potential as targets for therapeutic intervention.

### 2.3 Tumor angiogenesis

In the 1970s, Folkman proposed a hypothesis that was different from traditional theories, the tumor angiogenesis theory (91), which suggests that tumor growth depends on the formation of new blood vessels (92–94). He also first described the potential prospects of antiangiogenic cancer therapy. Tumor angiogenesis refers to the process of forming a new network of blood vessels during tumor growth. Under normal circumstances, angiogenesis is a highly regulated process that maintains the normal physiological function of tissues. However, in tumors, abnormal angiogenesis is a key process involved in tumor growth and metastasis. Tumor cells require a sufficient blood supply to obtain oxygen and nutrients, which are essential for their rapid proliferation and growth. Therefore, as the tumor volume increases, the existing blood vessels in the surrounding tissue are unable to meet its demands (95). To address this issue, tumor cells release a series of proangiogenic factors, such as vascular endothelial growth factor (VEGF) (96) and basic fibroblast growth factor (bFGF) (97), which stimulate the activation of endothelial cells in the surrounding tissue. Activated endothelial cells degrade the surrounding matrix by releasing enzyme substances such as metalloproteinases and proteases and migrating toward the tumor area. This process involves the regulation of multiple growth factors and signaling pathways, such as VEGF/vascular endothelial growth factor receptor (VEGFR) (98, 99), fibroblast growth factor (FGF)/fibroblast growth factor receptor (FGFR), Notch, and phosphatidylinositol 3-kinase (PI3K)/protein kinase B (PKB; also known as AKT) (100). The newly formed vascular structure needs to be stabilized through interactions with the extracellular matrix and other supportive cells in the surrounding tissue. This includes the recruitment of vascular smooth muscle cells and deposition of the extracellular matrix. Tumor cells can also recruit other types of cells, such as endothelial progenitor cells, stromal cells, and immune cells, by releasing chemokines. These cells can participate in the process of angiogenesis and provide support and regulation. During tumor angiogenesis, newly formed blood vessels often exhibit abnormal permeability, leading to the leakage of fluid and proteins into the surrounding tissue. This provides the tumor with increased nutrients and oxygen and creates a pathway for tumor metastasis.

In conclusion, the dynamic regulation of m^6^A modifications is significantly correlated with gene expression, and disruptions in the balance maintained by writers, erasers, and readers often lead to pathological conditions, ultimately resulting in tumorigenesis (17, 101). M^6^A is a widely occurring modification of both mRNAs and ncRNAs that participates in RNA splicing, translation, and stability regulation and influences the function of specific ncRNAs through epigenetic mechanisms (102). Research has demonstrated that the regulation of ncRNAs by m^6^A plays a crucial role in tumor initiation, metastasis, and angiogenesis (103–105). A substantial body of evidence indicates that ncRNAs mediate interactions between RNAs and between RNAs and proteins, regulating specific biological functions and thereby affecting cellular processes and contributing to tumor development and progression. Moreover, m^6^A and ncRNAs may exhibit synergistic effects in cancer therapy, with their regulatory mechanisms offering significant potential for clinical applications (106–108). In recent years, m^6^A has emerged as a promising biomarker for cancer detection and prevention, with its clinical potential in oncology becoming increasingly apparent. However, further in-depth research is necessary to elucidate the specific mechanisms and applications of m^6^A modifications in tumors (109–111). This research is expected to provide novel targeted strategies for cancer treatment and a theoretical foundation for the development of new anticancer therapeutics.

## 3 Direct and indirect effects of m^6^A on tumor angiogenesis

### 3.1 Direct regulation of m^6^A in tumor angiogenesis

#### 3.1.1 Writers act on tumor angiogenesis

RNA methylation is closely associated with tumor angiogenesis. As one of the core components of m^6^A modification, METTL3 plays a crucial role in m^6^A modification and has been identified as an oncogenic target in some hematological malignancies and solid tumors (112, 113). Currently, studies have elucidated the role of METTL3 in promoting cell proliferation and angiogenesis, and its role in promoting tumor cell proliferation and angiogenesis has been confirmed through a series of experiments. METTL3 plays a dual role in normal and leukemic myeloid cells by driving the translation of key genes to maintain acute myeloid leukemia (AML) cell proliferation and undifferentiation. Inhibition of METTL3 induces differentiation and apoptosis, presenting a viable therapeutic strategy (114). Experimental data indicate that in gastric cancer (GC), METTL3 promotes tumor angiogenesis and carcinogenesis by reducing the expression of ADAMTS9 (115). In myeloid leukemia and lung cancer (LC), inhibiting METTL3 has been shown to be a promising therapeutic strategy in the future (114, 116). Recent studies have also demonstrated that METTL3-mediated m^6^A modification can activate the PI3K/AKT/mammalian target of rapamycin (mTOR) pathway in ovarian cancer (OC) (117) and retinoblastoma (Rb) (118). These pathways play important roles in regulating protein synthesis, cell growth, and protein synthesis related to angiogenesis. They play crucial regulatory roles in tumor angiogenesis. Research has confirmed that in bladder cancer (BCa), METTL3 also regulates the PI3K/AKT pathway, which is involved in tumor angiogenesis (119). Moreover, METTL3-mediated m^6^A modification is essential for the activation of tie-2 receptor tyrosine kinase (TEK)/vascular endothelial growth Factor A (VEGFA)-mediated BCa progression and angiogenesis. In colorectal cancer (CRC), METTL3 upregulates plasminogen activator urokinase (PLAU) mRNA in a m^6^A-dependent manner and participates in the mitogen-activated protein kinase (MAPK)/extracellular signal-regulated kinase (ERK) pathway to promote tumor angiogenesis and metastasis (120). In head and neck squamous cell carcinoma and other types of tumor cells, interleukin-8 (IL-8) and VEGF are coexpressed and promote tumor growth, invasion, and angiogenesis (121). The MAPK/ERK pathway can regulate tumor angiogenesis by upregulating or downregulating the synthesis of IL-8 and VEGF. Research on glioblastoma (GBM) has revealed that METTL3 enhances the stability of BUD13 mRNA through m^6^A methylation (122). BUD13 overexpression enhances the stability of CDK12 mRNA and increases its expression. Subsequently, CDK12 phosphorylates MBNL1, ultimately promoting the proliferation, migration, invasion, and vasculogenic mimicry (VM) of GBM. The METTL3-induced transcription factor interferon regulatory Factor 5 (IRF5) promotes proliferation, migration, invasion, and angiogenesis in cervical cancer (CC) cells by upregulating the protein phosphatase 6 catalytic subunit (PPP6C) (123). In addition, as another important component of the m^6^A MTC, METTL14 also plays a role in malignant tumors (124–127). Studies have shown that m^6^A modification of TRAF1, which is dependent on METTL14, promotes sorafenib resistance by regulating apoptosis and angiogenesis pathways (128). Interestingly, in tongue squamous cell carcinoma (TSCC), METTL14 reduces the stability of basic leucine zipper ATF-like transcription Factor 2 (BATF2) through m^6^A modification, thereby promoting TSCC proliferation, migration, invasion, and angiogenesis (129).

In summary, the m^6^A writers METTL3 and METTL14 play important roles in the occurrence and development of tumors by regulating specific targets or pathways that affect tumor angiogenesis. These studies may provide new possibilities for the clinical treatment of tumors (**Table 1**).

**Table 1:** The regulatory role of key m^6^A members in tumor angiogenesis.

M^6^A Regulator	Cancer type	Role	Functional classification	Mechanism	References
Writer					
METTL3	GC	Oncogene	Promoting tumor growth *in vivo*	Regulating its target, ADAMTS9	(98)
	GC	Oncogene	Enhancing glycolysis and promoting angiogenesis contribute to the occurrence and metastasis of GC	Promotes the progression of GC through the METTL3/HDGF/GLUT4/ENO2 axis	(130)
	OC	Oncogene	Regulation of protein synthesis related to angiogenesis and cell growth	Activating the PI3K/AKT/mTOR pathway and phosphorylates related genes such as S6K1 and 4E-BP1	(101)
	Rb	Oncogene	Enhancing the proliferation, migration, and invasion of Rb cells while reducing cell apoptosis	Activating the PI3K/AKT/mTOR pathway	(102)
	BCa	Oncogene	Promoting the angiogenesis of BCa peripheral blood vessels is associated with cancer progression	Activating the PI3K/AKT pathway and TEK/VEGF-A	(103)
	CRC	Oncogene	Promoting tumor angiogenesis and metastasis	Upregulating PLAU mRNA promotes CRC cell angiogenesis through the PLAU/MAPK/ERK pathway	(104)
	HNSCC	Oncogene	Promoting the proliferation, migration, invasion, and angiogenesis of HNSCC cells	Regulating CDC25B mRNA	(105)
	GBM	Oncogene	Promoting the proliferation, migration, invasion, and vasculogenic mimicry of GBM cells	High expression of BUD13 enhances the expression of CDK12, and CDK12 phosphorylates MBNL1	(106)
	CC	Oncogene	Promotes tumor cell migration, invasion, and angiogenesis	Stabilizes IRF5 RNA, thereby upregulating PPP6C expression	(107)
METTL14	RCC	Oncogene	Promoting angiogenesis and metastasis of RCC	Upregulating the expression of TRAF1	(112)
	TSCC	Oncogene	Promotes cell proliferation, migration, invasion, and angiogenesis in TSCC	Inhibits BATF2 expression	(113)
WTAP	CRC	Oncogene	Promotes CRC progression and angiogenesis	Mediated by YTHDC1, activates the MAPK signaling pathway, increasing VEGFA expression	(131)
Eraser					
FTO	AML	Oncogene	Inhibiting ATRA-induced differentiation of AML cells	Regulating the expression of targets such as ASB2, RARA	(125)
	ICC	Tumor suppressor	Inhibiting non-adherent growth and migration of ICC cells and reducing FTO expression endogenously *in vitro* can decrease apoptosis of ICC cells	Regulating the RNA stability of target genes to control their expression, such as TEAD2	(126)
	CRC	Oncogene	Promotes colorectal cancer cell growth, metastasis, and angiogenesis	Stabilizes ZNF687 expression, activating the Wnt/β-catenin signaling pathway	(132)
Reader					
YTHDF2	HCC	Tumor suppressor	Inhibiting the proliferation, apoptosis, and angiogenesis of HCC cells	Inhibiting the overexpression of IL-11 and SERPINE2 promotes inflammation and vascular remodeling in HCC	(119)
		Oncogene	Promotes HCC cell immune evasion and angiogenesis	Recruits eIF3b to promote ETV5 translation, increasing PD-L1 and VEGFA expression	(133)
YTHDF3	BC	Oncogene	Promoting the interaction between cancer cells and brain endothelial cells and astrocytes, permeation across the blood-brain barrier, angiogenesis, and growth	Acting on specific targets, such as ST6GALNAC5, GJA1, EGFR, and VEGFA	(120)
IGF2BP2	CRC	Oncogene	Promoting the metastatic progression of CRC	Regulating the progression of CRC through interaction with METTL3	(105)
	LUAD	Oncogene	Promotes angiogenesis and metastasis in LUAD	Enhances FLT4 mRNA stability, activating the PI3K-Akt signaling pathway	(116)
IGF2BP3	Colon caner	Oncogene	Promoting angiogenesis in CRC cells	Recognizing and binding to VEGF mRNA, stabilizing and promoting its expression	(117)
	GC	Oncogene	Promoting angiogenesis in GC	Binding to the m^6^A site on HDGF mRNA, stabilizing its expression, and promoting HDGF secretion	(118)
	GC	Oncogene	Inhibiting hypoxia-induced migration and angiogenesis of GC cells	Directly binding to the specific m^6^A site in the coding region of HIF1a mRNA in GC cells, positively regulating the expression of HIF1a	(108)

#### 3.1.2 Readers act on tumor angiogenesis

Currently, an increasing number of studies have focused on the regulatory roles of m^6^A readers, such as insulin-like growth Factor 2 mRNA-binding protein 2 (IGF2BP2), IGF2BP3, YTH N6-methyladenosine RNA-binding protein 1 (YTHDF1), YTHDF2, and YTHDF3, in tumor occurrence, development, and angiogenesis. Research has shown that m^6^A readers play important roles in tumors such as CRC, GC, and BC brain metastasis. In CRC, METTL3 can promote CRC metastasis and progression via m^6^A-modified IGF2BP2 (134). Another study has shown that in CC, the m^6^A reader IGF2BP3 recognizes and binds to the m^6^A modification site on VEGF mRNA, promoting its stability and expression (135). VEGF is a vascular growth factor secreted by tumor cells or lymphocytes and has been shown to be a major factor in tumor angiogenesis. In studies of lung adenocarcinoma (LUAD), IGF2BP2 is transferred from LUAD cells to endothelial cells via exosomes. This transfer enhances the stability of FLT4 RNA, leading to the activation of the PI3K−Akt signaling pathway, which subsequently promotes angiogenesis and metastasis in cancer cells (136). IGF2BP2 is markedly overexpressed in AML and modulates crucial genes such as MYC, GPT2, and SLC1A5 through a m^6^A-dependent mechanism, thereby playing a pivotal role in glutamine metabolism and contributing to the pathogenesis and progression of AML (137). Therefore, IGF2BP3 can promote angiogenesis in CC cells by regulating VEGF. Experimental data have shown that the m^6^A reader IGF2BP3 binds to the m^6^A site on hepatoma-derived growth factor (HDGF) mRNA, maintaining its stability and promoting the secretion of HDGF (138), thereby promoting angiogenesis in GC tumors. IGF2BP3 also interacts with hypoxia-inducible factor-1a (HIF1a) and regulates the migration and angiogenesis of GC cells. YTHDF2 is one of the most efficient m^6^A readers and recognizes and distributes mRNAs containing m^6^A to processing bodies, thereby destabilizing the mRNA. Studies have shown that the overexpression of YTHDF2 in hepatocellular carcinoma (HCC) can inhibit tumor cell growth, whereas the knockout of the YTHDF2 gene promotes angiogenic sprouting in human umbilical vein endothelial cells (HUVECs). Silencing YTHDF2 can also promote tumor growth and metastasis in mouse models, and the key targets of YTHDF2 in HCC inflammation have been identified as IL11 and SERPINE2 (139). YTHDF3 expression is increased in BC brain metastases and is directly associated with reduced survival rates in BC patients without brain metastasis (140). It promotes the interaction between BC cells and brain endothelial cells and astrocytes, facilitating extravasation across the blood−brain barrier, angiogenesis, and growth.

In conclusion, the specific mechanisms of action of m^6^A readers in tumors are receiving increasing attention. Research has shown that m^6^A readers such as IGF2BP2, IGF2BP3, YTHDF2, and YTHDF3 are involved in tumor angiogenesis, further influencing the occurrence and development of tumors and potentially affecting the prognosis of patients with tumors to some extent (**Table 1**).

#### 3.1.3 Erasers act on tumor angiogenesis

To date, the demethylases discovered for m^6^A include mainly FTO and ALKBH5 (141, 142). The dynamic reversibility of RNA methylation is closely related to this process. However, relatively few studies have investigated the regulatory mechanisms of FTO and ALKBH5 in tumor development and angiogenesis. In pancreatic cancer (PC), ALKBH5 can regulate the tumor microenvironment, and its loss reduces the infiltration of CD8+ T cells in PC. Additionally, ALKBH5 can inhibit the motility of PCs by demethylating the lncRNA KCNK15-AS1 (143). In CRC, circ3823 is involved in the regulation of tumor cell growth, metastasis, and angiogenesis through the miR-30c-5p/TCF7 axis. The degradation rate of circ3823 may be regulated by the m^6^A recognition protein YTHDF3 and the demethylase ALKBH5. Therefore, ALKBH5 can indirectly regulate the expression of circ3823 to control the growth, metastasis, and angiogenesis of CRC (144). Research has shown that METTL14/ALKBH5 affects tumor growth and progression by regulating key cell cycle- and angiogenesis-related transcripts. In addition, the RNA-binding proteins HuR, METTL14/ALKBH5, and their target genes form a feedback loop, regulating each other’s expression in cancer cells and participating in the regulation of tumor occurrence and metabolism. Research has shown that METTL14/ALKBH5 affects tumor growth and progression by regulating key cell cycle- and angiogenesis-related transcripts. In addition, the RNA-binding proteins HuR and METTL14/ALKBH5 and their target genes form a feedback loop, regulating each other’s expression in cancer cells and participating in the regulation of tumor occurrence and metabolism (29). The FTO protein, which is associated with adiposity and obesity, can decrease the concentration of m^6^A in mRNA transcripts, thereby regulating the expression of target genes such as ASB2 and RARA. It inhibits ATRA-induced differentiation of AML cells and promotes the development of leukemia. Additionally, the FTO/m^6^A/myelocytomatosis oncogene (MYC)/(CCAAT/enhancer binding protein α) (CEBPA) signaling pathway plays a crucial role in leukemia (145). In patients with intrahepatic cholangiocarcinoma (ICC), the expression level of FTO is lower (146). Patients with low FTO expression are more likely to be CD34 positive (indicating microvessel density), suggesting that the expression of FTO may regulate tumor angiogenesis. However, further research is needed to elucidate the specific mechanism involved.

In summary, m^6^A demethylases interact with m^6^A writers and readers, influencing the process of m^6^A methylation and participating in the regulation of tumor angiogenesis. Therefore, m^6^A demethylases have the potential to become precise regulatory targets for tumor angiogenesis, playing a role in promoting or inhibiting tumor growth (**Table 1**).

### 3.2 Indirect effects of m^6^A on tumor angiogenesis: interactions with ncRNAs

#### 3.2.1 MiRNAs and m^6^A in tumor angiogenesis

**Figure 2:**
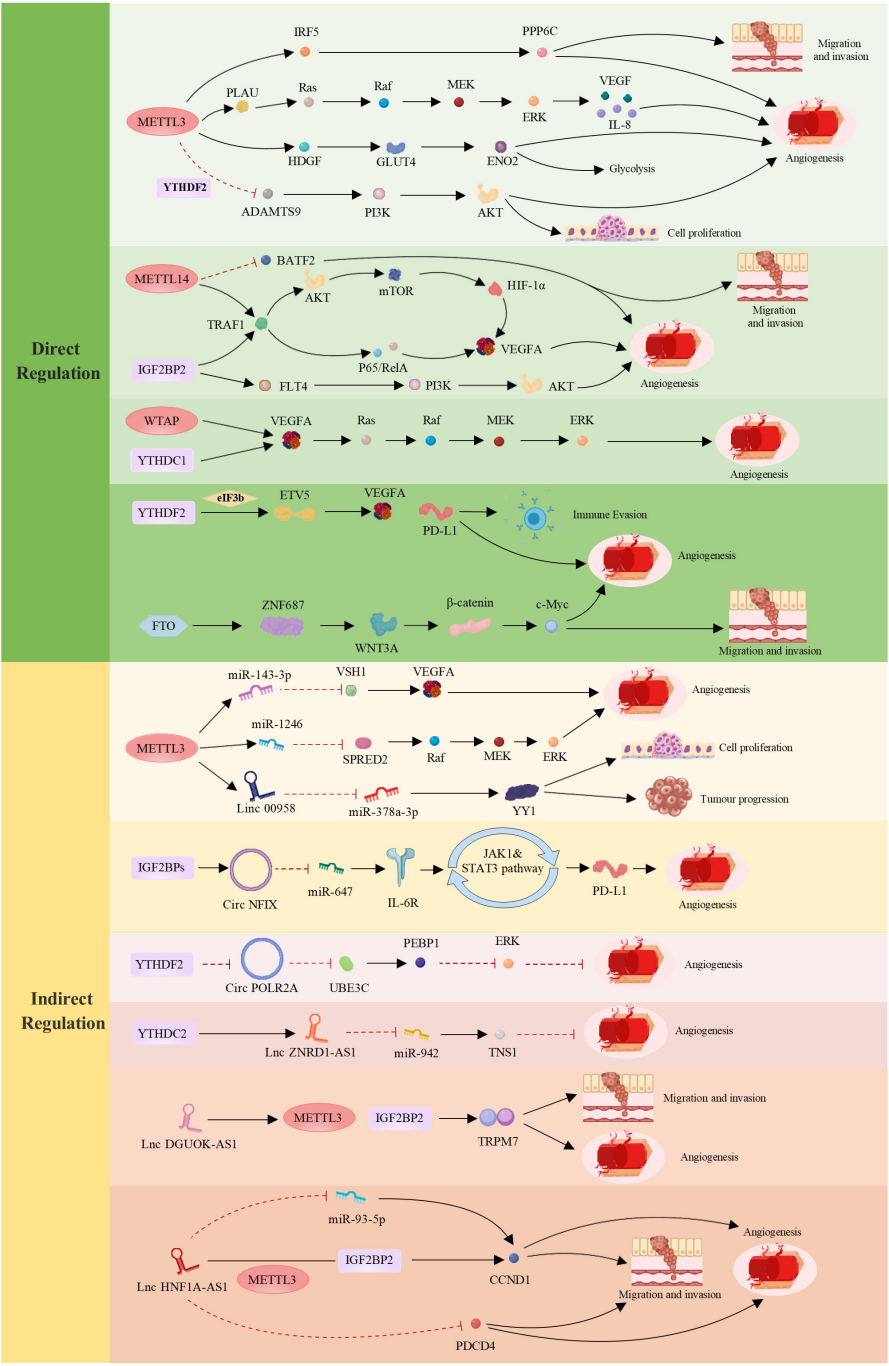
Regulatory roles of m^6^A in tumor angiogenesis and development—direct and indirect mechanisms. The direct regulation of m^6^A involves METTLs, ALKBHs, YTHDFs, IGF2BPs, YTHDCs and FTO, which influence specific targets and cellular signaling pathways, thereby modulating tumor angiogenesis and cancer progression. Indirect regulation of m^6^A occurs through interactions between the three main components of its “life cycle” and ncRNAs (including miRNAs, long noncoding RNAs (lncRNAs), and circular RNAs (circRNAs)), collectively mediating tumor cell proliferation, migration, angiogenesis, and immune evasion. These mechanisms play crucial roles in various tumors, profoundly impacting tumor biology. In the figure, red indicates inhibitory effects, whereas black indicates promoting effects. (Created with MedPeer (www.medpeer.cn)).

MiRNAs are a class of evolutionarily conserved noncoding small RNA molecules with lengths of approximately 20–24 nucleotides that play a role in regulating gene expression. Many studies have shown that miRNAs exert their biological functions by regulating the translation process of downstream genes (147). In tumors such as LC, endometrial cancer (EC), and BC, miRNAs regulate the expression of genes related to angiogenesis to influence tumor growth and metastasis. For example, in LC, miR-320b suppresses angiogenesis and tumor growth by downregulating the expression of IGF2BP2 and thymidine kinase 1 (TK1) (148). In LC, METTL3 promotes the maturation of miR-143-3p through methylation and targets vasohibin-1 (VASH1) to inhibit its expression, thereby affecting angiogenesis (149). In breast cancer (BC) brain metastasis, YTHDF3 enriches transcripts associated with metastasis and promotes the interaction between tumor cells and other cells in the tumor microenvironment, facilitating angiogenesis and metastasis (140). In CRC, overexpression of METTL3 leads to the methylation of pri-miR-1246 and promotes its maturation, which is positively correlated with tumor metastasis (150). These findings indicate that miRNAs play crucial roles in tumor angiogenesis and metastasis, further elucidating the regulatory mechanisms of miRNAs in tumor initiation and progression (**Figure 2**).

In conclusion, with in-depth research on m^6^A modification and miRNA regulatory mechanisms, we expect to discover more important mechanisms of mutual regulation between m^6^A modification and miRNAs in cancer initiation and progression. These findings will contribute to a better understanding of the biological characteristics of tumors and provide new targets and insights for the development of novel anticancer treatment strategies (**Table 2**).

**Table 2:** NcRNAs and m^6^A modifications are involved in the regulatory mechanisms of tumor angiogenesis.

M^6^A regulator	NcRNA	Cancer	Biological function and mechanism	References
Writer				
METTL3	MiR-143-3p	LC	The miR-143-3p/VASH1 axis may be mediated by METTL3 in an m^6^A-dependent manner by activating the ability of VEGFA to express and facilitate tube formation	(129)
	Pri-miR-1246	CRC	Overexpression of m^6^A writer METTL3 methylated pri-miR-1246, further promoted the maturation of pri-miR-1246, and was positively correlated with tumor metastasis	(134)
	LINC00958	BC	LINC00958 is controlled by METTL3 and promotes the occurrence of BC by regulating the miR-378a-3p/YY1 axis	(145)
	LncRNA DGUOK-AS1	NSCLC	Enhancement of TRPM7 mRNA stability through METTL3 and IGF2BP2 modifications promotes cell proliferation, migration, invasion, and angiogenesis	(147)
METTL14	LNC942	BC	LNC942 promotes BC cell proliferation and progression by regulating METTL4-mediated m^6^A methylation	(144)
Eraser				
ALKBH5	LncRNA KCNK15-AS1	PC	ALKBH5 reduces infiltration of CD8+ T cells in PC, and ALKBH5 can inhibit PC movement by demethylating lncRNA KCNK15-AS1	(123)
	LncRNA GAS5-AS1	CC	LncRNA GAS5-AS1 initiates ALKBH5-dependent m^6^A demethylation in CC, thereby inhibiting the proliferation, migration and invasion of CC cells	(146)
	Circ3823	CRC	Circ3823 promotes CRC growth, metastasis and angiogenesis through the circ3823/miR-30c-5p/TCF7 axis; the degradation rate of circ3823 is regulated by the m^6^A recognition protein YTHDF3 and the demethylase ALKBH5	(124)
Reader				
IGF2BPs	CircNFIX	OC	Inhibiting miR-647 increases IL-6R expression, activates the JAK1/STAT3 signaling pathway, upregulates PD-L1 expression, and promotes immune evasion and angiogenesis in ovarian cancer cells	(149)
IGF2BP2	MiR-320b	LC	Suppresses angiogenesis and tumor growth by downregulating IGF2BP2 and TK1 expression	(129)
	LncRNA HNF1A-AS1	CRC	IGF2BP2 and METTL3 competitively bind miR-93-5p to promote CCND1 expression, enhancing cancer cell proliferation, migration, and angiogenesis, while also inhibiting PDCD4 to facilitate cell cycle progression	(148)
YTHDF2	CircPOLR2A	cRCC	The m^6^A interpreter YTHDF2 can regulate the expression of circPOLR2A, which in turn regulates tumor angiogenesis	(151)
YTHDC2	LncRNA ZNRD1-AS1	LC	Inhibition of LC cell proliferation, migration, and angiogenesis via regulation of the miR-942/TNS1 axis	(149)

#### 3.2.2 LncRNAs and m^6^A in tumor angiogenesis

LncRNAs are ncRNA molecules that are longer than 200 nucleotides (152). Research has shown that lncRNAs play crucial regulatory roles in various biological processes, including cell proliferation (153, 154), differentiation, apoptosis (155), migration, and invasion (156). LncRNAs can form complementary double-stranded structures with the transcripts of protein-coding genes, interfere with mRNA splicing, regulate protein activity, and serve as precursor molecules for small RNAs such as miRNAs and piRNAs (157). Therefore, lncRNAs play important roles in regulating gene expression (151, 158). In tumors such as BC, CC, and CRC, different lncRNAs are involved in tumor initiation and progression through various mechanisms. For example, in BC, the lncRNA TDRG1 promotes the proliferation, invasion, and metastasis of BC cells through the miR-214-5p/recombinant chloride intracellular channel protein 4 (CLIC4) axis (159). LNC942 regulates METTL14-mediated m^6^A methylation to promote BC cell proliferation and progression (160). LINC00958, which is controlled by METTL3, promotes the occurrence of BC through the regulation of the miR-378a-3p/YY1 axis (161). Research has confirmed that the lncRNA GAS5-AS1 can initiate ALKBH5-dependent m^6^A demethylation in CC, thereby inhibiting the proliferation, migration, and invasion of CC cells (162). The evidence indicates that the lncRNA DGUOK-AS1 is markedly overexpressed in non-small cell lung cancer (NSCLC) tissues and cells. It facilitates NSCLC cell proliferation, migration, invasion, and angiogenesis by increasing the stability of transient receptor potential melastatin 7 (TRPM7) mRNA through METTL3-mediated and IGF2BP2-mediated modifications (163). In addition, the m^6^A reader YTHDF3 promotes the progression of CRC by facilitating the degradation of the m^6^A-modified lncRNA GAS5-AS1. Notably, the lncRNA HNF1A-AS1 facilitates the progression of the CRC cell cycle by upregulating CyclinD1 (CCND1) and inhibiting programmed cell death 4 (PDCD4). HNF1A-AS1 stabilizes CCND1 mRNA through its interaction with IGF2BP2, a process further enhanced by METTL3-mediated m^6^A modification. Additionally, HNF1A-AS1 competes with miR-93-5p, leading to increased CCND1 expression. This upregulation promotes CRC cell proliferation, migration, and angiogenesis, thereby driving tumor progression (164). Xiangrui Meng et al. reported that m^6^A-mediated overexpression of LINC00857 promotes the progression and occurrence of PC through the regulation of the mir-150-5p/E2F3 axis, which is closely associated with PC growth and angiogenesis (143). The m^6^A modification mediated by YTHDC2 enhances the stability of ZNRD1-AS1. In turn, ZNRD1-AS1 suppresses the proliferation, migration, and angiogenesis of LC cells by modulating the miR-942/tensin 1 (TNS1) axis (165) (**Figure 2**).

In conclusion, these findings demonstrate the significant role of lncRNAs in tumor initiation and progression, which are closely associated with tumor growth, invasion, and angiogenesis. In recent years, many m^6^A-modified lncRNAs have been discovered, and they regulate gene expression and function through a complex series of mechanisms. We believe that in the future, more m^6^A-modified lncRNAs regulated by other mechanisms will be discovered (**Table 2**).

#### 3.2.3 CircRNAs and m^6^A in tumor angiogenesis

CircRNAs are a type of ncRNA that exist in organisms. They are characterized by the absence of a 5’ cap and a 3’ poly(A) tail, instead of forming a covalently closed loop structure (166). Most circRNAs are generated through back-splicing of exons, whereas a small portion is derived from introns. Due to their closed circular structure (167), circRNAs are more stable than linear RNAs and are resistant to degradation by exonucleases. Additionally, circRNAs exhibit species-specific, tissue-specific, and time-specific expression patterns (168, 169). It also shares some sequence conservation (170) and can exert regulatory functions at the transcriptional or posttranscriptional level (171). Research has shown that the majority of circRNAs are noncoding, but few can be translated into peptides. Currently, circRNAs have been found to have functions such as acting as miRNA sponges (172, 173), regulating protein binding and gene transcription, and encoding peptides. Many studies have demonstrated that circRNAs play important roles in the growth, development, stress response, and disease progression of organisms. In recent years, interest in the impact of m^6^A modification on circRNAs in tumor initiation, invasion, and angiogenesis has increased. For example, studies in clear cell kidney cancer (cRCC) have shown that circPOLR2A can downregulate the protein level of recombinant phosphatidylethanolamine binding protein 1 (PEBP1), thereby activating the ERK signaling pathway. Activation of the ERK pathway plays a crucial role in cancer angiogenesis. Therefore, circPOLR2A, an oncogene in cRCC, accelerates the proliferation, migration, invasion, and angiogenesis of cRCC while inhibiting apoptosis (174). In addition, in CRC, circ3823 plays a significant role in tumor growth, metastasis, and angiogenesis (144). Circ3823 can inhibit miR-30c-5p and promote the expression of downstream targets MYC and CCND1, which further promotes CRC growth, metastasis, and angiogenesis through the circ3823/miR-30c-5p/TCF7 axis. We also detected m^6^A modifications on circ3823. The degradation rate of circ3823 is regulated by the m^6^A recognition protein YTHDF3 and the demethylase ALKBH5. Therefore, precise regulation of circ3823 by m^6^A modification is involved in tumor angiogenesis and contributes to tumor initiation and progression. In OC, circNFIX, which is activated by IGF2BPs (IGF2BP1/2/3), inhibits miR-647, resulting in the upregulation of IL-6R expression. This upregulation subsequently triggers the activation of the JAK1/STAT3 signaling pathway, leading to increased PD-L1 expression. These molecular changes facilitate immune evasion and angiogenesis in OC cells (165) (**Figure 2**).

In summary, precise regulation of circRNAs by m^6^A modification plays a crucial role in cancer progression and may provide new insights into tumor initiation, development, and precision therapy. However, relatively few examples of the regulation of circRNAs by m^6^A modification are currently available, and further in-depth research on these modifications is needed. We believe that in the future, more studies on the mutual regulatory mechanisms between circRNAs and m^6^A modifications will emerge (**Table 2**).

## 4 Clinical translational potential of m^6^A modifications in oncology

### 4.1 Characterized m^6^A inhibitors

Given the important role of m^6^A regulatory proteins in various diseases, small-molecule inhibitors or agonists that target dysregulated m^6^A regulators may be promising candidates for disease treatment, especially cancer therapy. METTL3, the most extensively studied m^6^A methyltransferase to date, has attracted considerable attention from researchers. In tumor cells, aberrant expression and activity of METTL3 can lead to changes in m^6^A modification levels, thereby influencing tumor initiation, progression, and metastasis. Studies have shown that METTL3 inhibitors exhibit significant anticancer effects in various tumor models. For example, STM2457 selectively inhibits METTL3 methyltransferase activity, reducing m^6^A levels in acute AML cells, inhibiting their proliferation, and inducing apoptosis (175). RSM3 interferes with METTL3’s catalytic activity, reducing m^6^A modifications and showing antitumor potential in AML cell lines (176). UZH1a, a specific inhibitor, significantly affects viral replication and cell survival in EBV-positive Akata cells by inhibiting METTL3 methyltransferase activity (177). This discovery highlights METTL3 as a potential therapeutic target in EBV-associated diseases such as nasopharyngeal carcinoma (NPC), Hodgkin’s lymphoma, and GC, indicating the clinical application prospects of UZH1a. Quercetin, a natural compound, inhibits METTL3 activity, reduces m^6^A modifications, and, thus, inhibits tumor cell proliferation and migration, providing new insights into the development of natural product-based anticancer drugs (178).

YTHDF1 recognizes and binds to m^6^A-modified RNA, regulating the translation of various cancer-related genes and playing crucial roles in tumor initiation, progression, and therapeutic resistance. Studies have shown that RUVBL1/2 interferes with the RNA binding activity of YTHDF1, reducing the translation of cancer-related genes and inhibiting tumor cell proliferation and invasion, demonstrating significant antitumor effects in CRC (179). IGF2BP2, another m^6^A reader protein, binds to m^6^A-modified RNA, regulating its stability and translation. In AML, IGF2BP2 regulates key targets in the glutamine metabolic pathway (e.g., MYC, GPT1, and SLC5A6) in a m^6^A-dependent manner, promoting the development and self-renewal of leukemia stem/initiation cells. The small-molecule compound CWI1-2 inhibits IGF1BP2, showing promising antileukemic effects both *in vitro* and *in vivo* (137). In T-cell acute lymphoblastic leukemia (T-ALL), IGF2BP2 is highly expressed. Studies have confirmed that JX5 can inhibit the binding of IGF2BP2 to NOTCH1, thereby inactivating NOTCH1 signaling in T-ALL. However, the off-target effects and toxicity of JX5 require further investigation (180).

ALKBH5 demethylates m^6^A modifications, maintaining the stability of specific RNAs and regulating specific gene splicing processes, thereby affecting cancer cell proliferation and migration. Studies have shown that the imidazobenzoxazin-5-thione MV1035 inhibits the catalytic activity of ALKBH5 by competitively binding with 2-oxoglutarate (2OG), significantly reducing the migration and invasiveness of the U87 GBM cell line. ALKBH5, as a potential target for cancer therapy, holds significant research value (181). IOX3, a small molecule inhibitor, significantly inhibits the demethylation activity of ALKBH5 *in vitro* (130). As an ALKBH5 inhibitor, the mechanisms of IOX3, which is related to tumor angiogenesis, need further investigation to provide new drug directions for cancer treatment. ALK-04, an effective ALKBH5-specific inhibitor, has demonstrated good antitumor potential in a B16 melanoma mouse model. Future research should explore the application of ALK-04 in different cancer types and investigate its combined effects with other therapies to increase overall cancer treatment efficacy (182).

Since FTO is one of the most extensively studied regulatory proteins involved in m^6^A modification, an in-depth understanding of its dysregulation has facilitated the development of small-molecule compounds that target it. Rhein, the first reported FTO inhibitor, competitively blocks the recognition of m^6^A substrates by FTO, but it lacks selectivity (183, 184). In a mouse GBM model, MA2 binds to the active surface of FTO, inducing m^6^A methylation, reducing GBM stem cell proliferation *in vitro*, and exerting good antitumor effects (131, 185). FTO-04 and FTO-43 are effective inhibitors of FTO. FTO-04 has been reported to impair the self-renewal ability of GBM stem cells, thereby inhibiting tumor progression (132). However, FTO-43 is a novel FTO inhibitor with nanomolar potency and high selectivity. Compared with the homologous m^6^A RNA demethylase ALKBH5, FTO-43 exhibits remarkable selectivity and effectively inhibits the Wnt/PI3K-Akt signaling pathway. Experimental results demonstrate that FTO-43 has potent antiproliferative effects in models of GBM, acute myeloid leukemia, and GC, with efficacy comparable to that of the clinically used chemotherapeutic agent 5-fluorouracil (5-FU) (186). As a novel immunosuppressant, FTO-43 has significant potential to greatly improve therapeutic outcomes in clinical applications. *In vitro* experiments indicate that the FTO inhibitor CS1 significantly inhibits cell proliferation and induces apoptosis in CRC-related cell lines and that it can downregulate the Akt/mTOR signaling pathway (187). The research team developed two FTO inhibitors, FB23 and FB23-2, and used CRISPR-Cas9 gene editing to create stable FTO knockout (KO) AML cell lines to assess the efficacy of these inhibitors. The results revealed that the antiproliferative effect of FB23-2 was significantly reduced in FTO-KO cells compared with wild-type cells, indicating that the mechanism of FB23-2 activity relies primarily on FTO inhibition. FB23-2 suppresses AML cell proliferation and induces differentiation and apoptosis by increasing m^6^A levels, demonstrating its potential as a therapeutic strategy for AML. The CRISPR-Cas9-generated FTO-KO model further validated FTO as a viable therapeutic target and provided new insights into its role in AML pathogenesis (188). Dac51 inhibits FTO-mediated tumor cell glycolytic activity, enhancing CD8^+^ T-cell function and inhibiting solid tumor growth (133). R-2-Hydroxyglutarate (R-2HG) attenuates aerobic glycolysis in leukemia by targeting the FTO/m^6^A/PFKP/LDHB axis, thereby modulating disease progression. These findings underscore the potential of potent FTO inhibitors as epigenetic modulators and metabolic targets for therapeutic intervention in cancer treatment (189). Recently, a successfully synthesized GSH bioimprinted nanocomposite loaded with an FTO inhibitor (GNPIPP12MA) was proven to inhibit leukemogenesis by targeting the FTO/m^6^A pathway and synergizing with GSH depletion (190). Notably, in addition to antitumor therapy, FTO inhibitors have also attracted attention for other diseases. For example, entacapone has been reported as a potential FTO inhibitor for the clinical treatment of metabolic syndromes such as obesity and diabetes (191).

The biological study of m^6^A modifications is at a critical stage of translational application, urgently requiring the discovery and development of chemical probes and active lead compounds. The development of highly selective chemical inhibitors targeting m^6^A writers, readers, and erasers has greatly advanced the understanding of the biological functions of m^6^A and demonstrated its feasibility as a therapeutic target. These findings lay the foundation for new models in basic research and cancer therapy in the field of RNA epigenetics.

### 4.2 Prospective m^6^A inhibitory targets: emerging directions in clinical oncology

#### 4.2.1 Digestive system cancers

##### 4.2.1.1 Gastric cancer

GC is the fifth most common gastrointestinal malignancy and the third leading cause of cancer-related death worldwide (192, 193). Due to the late diagnosis of most GC patients at an advanced stage of malignant spread and metastasis, the prognosis for these patients is generally poor. The dissemination and metastasis of cancer involve various biological processes, such as cell growth, migration, invasion, and angiogenesis. Therefore, there is an urgent need to identify biomarkers for GC diagnosis and prognostic evaluation, as well as therapeutic targets. A study involving *in vitro* formation and CAM experiments revealed that METTL3 may inhibit the m^6^A modification of its target gene ADAMTS9 through the ADAMTS9-mediated PI3K/AKT pathway and prevent its transcription in a YTHDF2-dependent manner. These findings suggest that ADAMTS9 could be a novel potential therapeutic target for the treatment of GC carcinogenesis and angiogenesis involving METTL3 (115). This study reveals the pathological role and molecular mechanism of METTL3, further supporting its potential as a prognostic biomarker and therapeutic target for GC. Recently, Jiang et al. reported that knockout of the m^6^A reader IGF2BP3 can inhibit hypoxia-induced GC cell migration and angiogenesis by regulating HIF1a. Experimental results indicate that HIF1a is a target of IGF2BP3 and that IGF2BP3 positively regulates the expression of HIF1a in GC cells by directly binding to specific m^6^A sites in the HIF1a mRNA coding sequence (CDS) (138). Under hypoxic conditions, IGF2BP3 promotes angiogenesis in GC cells by upregulating HIF1a. These findings may provide new therapeutic targets for the clinical treatment and prognosis of GC. Wang et al. reported that METTL3-mediated m^6^A modification maintains the expression of HDGF through IGF2BP3-dependent mRNA stability. Increased secretion of HDGF promotes tumor angiogenesis and glycolysis, thereby accelerating the malignant progression of GC and indicating a poor prognosis. These findings reveal that the METTL3/HDGF/GLUT4/ENO2 axis promotes the occurrence and metastasis of GC by enhancing glycolysis and angiogenesis (194).

In conclusion, the development and progression of GC, as well as angiogenesis, are intricately linked to m^6^A RNA methylation, which is precisely regulated by m^6^A-associated proteins. Nonetheless, a deeper investigation into inhibitors targeting m^6^A-related proteins involved in the mechanistic pathways of GC is warranted. These results will provide significant support for clinical drug development.

##### 4.2.1.2 Colorectal cancer

CRC is one of the most common malignant tumors in the gastrointestinal tract (195). It often lacks obvious early symptoms and is typically diagnosed in the middle to late stages of tumor development. As a result, nearly one million people are diagnosed with CRC each year, with a mortality rate of 33%, ranking it as the second leading cause of cancer-related deaths. Clinical data show that early detection, diagnosis, and treatment can lead to a 5-year survival rate of 90% for CRC patients (196). However, for patients with advanced metastasis, the 5-year survival rate decreases to only 8%. Therefore, it is crucial to study the targets involved in the occurrence, treatment prognosis, and inhibition of angiogenesis in CRC (197). Recent studies have revealed that METTL3 upregulates PLAU mRNA in a manner dependent on m^6^A modification and is involved in the MAPK/ERK pathway, promoting angiogenesis and metastasis in CRC. Elevated expression of METTL3 in CRC tissues has been associated with lower survival rates during cancer metastasis (134). Furthermore, in describing the epigenetic characteristics of CRC metastasis to the liver and lungs, METTL14 promotes cancer cell proliferation and metastasis by facilitating processes such as epithelial−mesenchymal transition (EMT) and protein phosphorylation through downstream targets such as the lncRNA RP11 and microRNAs (198). This significantly enhances the ability of CRC to metastasize to distant organs, leading to poor patient prognosis. WTAP modifies VEGFA mRNA through m^6^A, which is mediated by YTHDC1 and activates the MAPK signaling pathway. This process leads to increased VEGFA expression, promoting the progression and angiogenesis of CRC. These findings suggest that m^6^A inhibitors could be used in clinical treatments (199). In addition, it has been reported that the m^6^A reader IGF2BP3 can regulate the cell cycle and angiogenesis in CRC cells. Further research data suggest that IGF2BP3 inhibits the expression of VEGF by reading and promoting the decay of m^6^A-modified mRNAs, thereby suppressing angiogenesis in CC tumor cells and inhibiting tumor growth (135). A research team reported that, in CRC tissues and cell lines, FTO-mediated regulation of ZNF687 promotes tumor growth, metastasis, and angiogenesis through the Wnt/β-catenin pathway. The development of FTO inhibitors offers a new perspective for potential CRC treatment strategies (200).

As a result, in CRC, METTL3, METTL14, WTAP, IGF2BP3, YTHDC1, and FTO orchestrate tumor angiogenesis through diverse pathways and mechanisms, thereby affecting the initiation, progression, and prognosis of CRC. Notably, the writers, erasers, and readers of m^6^A collaboratively regulate CRC. Hence, the future application of m^6^A inhibitors in CRC and the equilibrium required for their use merit comprehensive investigation and deeper exploration.

##### 4.2.1.3 Hepatocellular carcinoma

HCC is a highly lethal primary liver cancer and one of the most common malignant tumors worldwide (201). Angiogenesis plays a crucial role in the growth and metastasis of HCC, making the inhibition of angiogenesis an important therapeutic target for HCC (202). Recent studies have shown that the m^6^A methyltransferase METTL3 is significantly associated with the formation of VM and the expression of VM-related markers and is closely related to poor prognosis in HCC. Additionally, the YAP1 protein promotes VM and malignant progression in HCC through a m^6^A-dependent mechanism in the Hippo signaling pathway. Therefore, METTL3 and YAP1 may serve as potential targets for the targeted inhibition of VM in the treatment of HCC (203). Recent research has demonstrated that inhibiting YTHDF2 can suppress immune evasion and angiogenesis in HCC through the ETS variant transcription Factor 5 (ETV5)/PD-L1/VEGFA axis. These findings indicate that targeting YTHDF2 may serve as an effective therapeutic strategy for HCC, providing a promising new avenue for combination treatments (204).

In summary, METTL3 and YTHDF2 may act as “oncogenes” in HCC, facilitating tumor angiogenesis and malignant progression. However, further rigorous research is needed to elucidate the precise regulatory mechanisms of m^6^A modification in HCC. These identified m^6^A-related proteins are pivotal targets for the development of clinical m^6^A inhibitors.

#### 4.2.2 Urinary system cancers

##### 4.2.2.1 Renal cell carcinoma

Localized renal cell carcinoma can be treated with surgery, whereas metastatic renal cancer is usually resistant to conventional radiotherapy and chemotherapy (192, 205). In recent years, there has been preliminary progress in inhibiting tumor development and metastasis by targeting the angiogenesis of RCC with drugs (206). A study of RCC revealed that tumor necrosis factor receptor (TNFR)-associated Factor 1 (TRAF1) is closely related to cancer cell angiogenesis and apoptosis. The overexpression of TRAF1 significantly enhances angiogenesis, whereas the downregulation of TRAF1 inhibits angiogenesis. The experimental results indicate that m^6^A methyltransferase METTL14-mediated m^6^A modification enhances the stability of TRAF1 mRNA and increases TRAF1 levels through an IGF2BP2-dependent mechanism. The increased expression of TRAF1 subsequently contributes to the activation of downstream antiapoptotic and proangiogenic pathways in sunitinib-resistant cells, thereby promoting angiogenesis and metastasis in RCC. In sunitinib-resistant RCC cells, the expression of TRAF1 can be effectively suppressed by regulating METTL14, thereby inhibiting the angiogenesis signaling pathway and activating the apoptosis signaling pathway (128). This approach can effectively control the drug resistance and metastasis of cancer cells in the clinical treatment of RCC (207).

In summary, current research has demonstrated that m^6^A modification plays a pivotal role in the clinical treatment and drug resistance of renal cell carcinoma (RCC), providing new hope for patients. We anticipate that future discoveries of relevant regulatory mechanisms and m^6^A-specific inhibitors will significantly increase the effectiveness of targeted therapies in overcoming drug resistance in RCC.

##### 4.2.2.2 Bladder cancer

Currently, METTL3-catalyzed m^6^A RNA methylation is widely recognized as a key epigenetic regulatory process for tumorigenic characteristics in various cancer cell lines, including BCa. Kyoto Encyclopedia of Genes and Genomes (KEGG) transcriptome sequencing results revealed a close association between METTL3 and tumor angiogenesis. M^6^A modification mediated by METTL3 is essential for the activation of tumor progression and angiogenesis mediated by TEK/VEGFA. In BCa, METTL3 regulates the PI3K/AKT pathway associated with tumor angiogenesis and promotes tumor angiogenesis by modulating TEK and VEGFA. METTL3 plays a crucial role in driving the progression of BCa by promoting angiogenesis around tumor cells. It affects bladder malignancies through the METTL3-TEK-VEGFA-CD31/CD34 pathway (119).

Overall, in the clinical treatment of BCa, targeting METTL3 could present novel therapeutic strategies to overcome chemotherapy and immunotherapy resistance driven by tumor angiogenesis, thereby improving the clinical outcomes of patients. The future development of more precise and highly specific METTL3 inhibitors promises to bring new hope to this patient population.

#### 4.2.3 Epidermal cancers

##### 4.2.3.1 Head and neck squamous cell carcinoma

Head and neck squamous cell carcinomas (HNSCCs) are a group of malignant tumors that occur in the head and neck region and account for approximately 90% of all head and neck tumors. They include tumors in the neck, oral and maxillofacial region, and otolaryngology (208, 209). Women are more susceptible to this disease. HNSCCs rank eighth in terms of the global incidence of malignant tumors and twelfth in terms of mortality. The 5-year survival rate is approximately 50%, and there has been no significant improvement in the past 20 years. This may be attributed to the late-stage detection of cancer and a lack of effective therapeutic targets. A recent study revealed that overexpression of METTL3 in HNSCC can promote cell proliferation, migration, invasion, and angiogenesis. METTL3 can mediate m^6^A modification of CDC25B mRNA, thereby promoting the malignant progression of HNSCC (121).

In conclusion, METTL3 is a promising prognostic biomarker and therapeutic target for HNSCC. This finding has the potential to address the high mortality rate and the current paucity of effective therapeutic targets in HNSCC. Consequently, comprehensive research into the regulatory mechanisms of m^6^A modification in HNSCC and the development of highly specific clinical inhibitors are crucial.

#### 4.2.4 Female reproductive system cancers

##### 4.2.4.1 Breast cancer

According to the latest data from the International Agency for Research on Cancer (IARC) in 2018, BC has the highest incidence rate among female cancers globally, accounting for 24.2% of all female cancer cases. Among these cases, 52.9% occur in developing countries (210). Although the expression and regulatory patterns of target genes associated with BC have been widely reported, the posttranscriptional regulatory mechanisms of gene expression during BC metastasis remain unclear. This lack of understanding can have implications for tumor treatment and prognosis. Recent studies have shown that the reader protein YTHDF3, which is involved in the epigenetic regulation process, plays a crucial role in BC and its brain metastasis. YTHDF3 enhances the translation of m^6^A-enriched transcripts such as ST6GALNAC5, gap junction 1 (GJA1), and EGFR, promoting the interaction between BC cells and brain endothelial cells and astrocytes. This interaction leads to extravasation across the blood−brain barrier, angiogenesis, and growth. YTHDF3-mediated m^6^A modification plays a crucial role in the development of brain metastasis in BC, which relies on the enhanced m^6^A methylation status and translation efficiency of target transcripts (140).

Consequently, YTHDF3 plays a crucial role in angiogenesis during the brain metastasis of BC. We believe that comprehensive research on m^6^A modification may provide new evidence and novel strategies for the clinical treatment and drug development of BC metastasis in the future.

**Figure 3:**
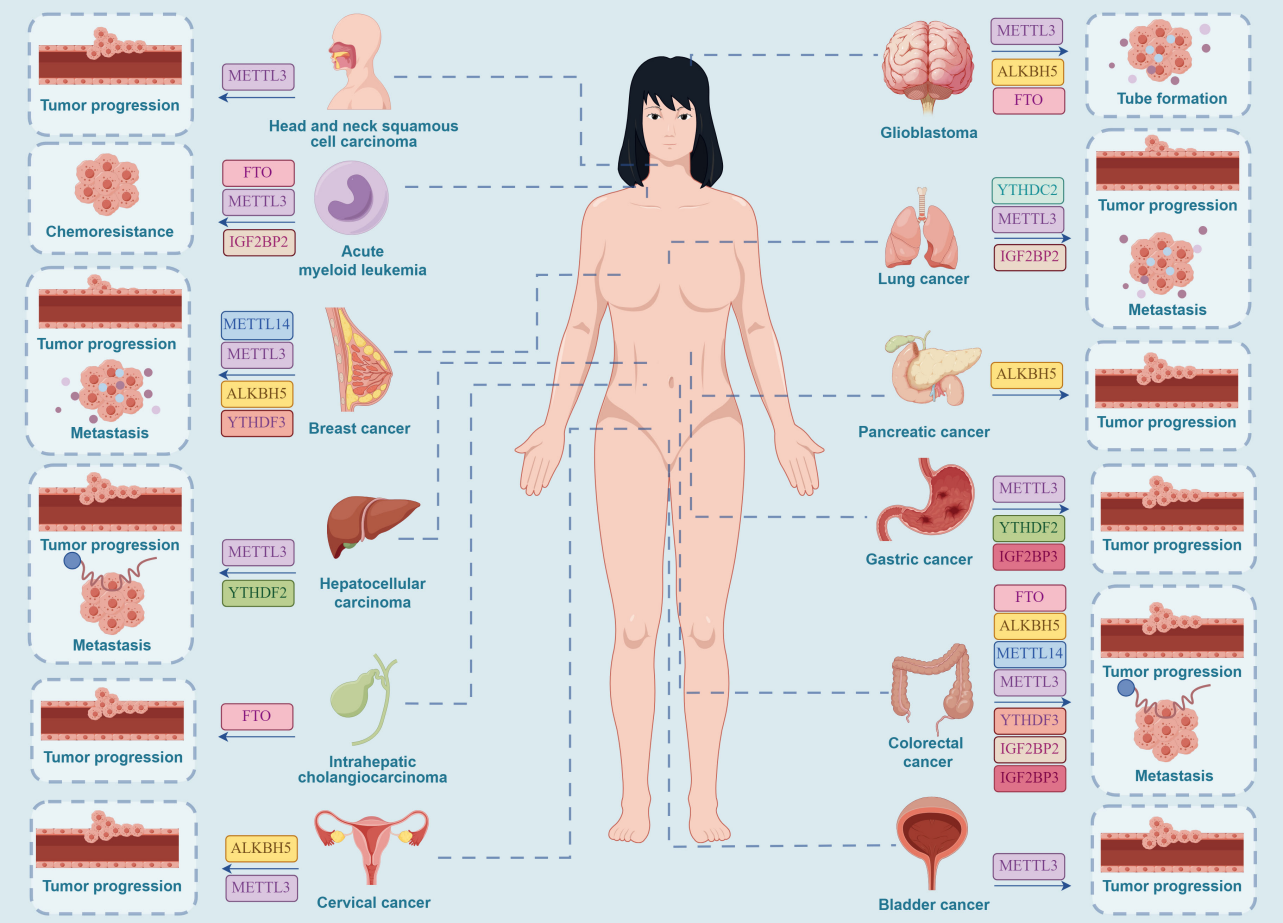
Effective targets of m^6^A inhibitors in clinical oncology therapy. METTL3 can serve as a clinical inhibitory target for a range of cancers, including HNSCC, AML, GC, BC, CC, HCC, GBM, LC, CRC, and BCa. METTL14 can serve as a clinical inhibitory target for brain metastases of BC and CRC. ALKBH5 can serve as a clinical inhibitory target for CC in females and for GBM, PC, CRC, and brain metastases of BC. FTO can serve as a clinical inhibitory target for AML, GBM, CRC, and ICC. YTHDF2 can serve as a clinical inhibitory target for HCC and GC. YTHDF3 can serve as a clinical inhibitory target for brain metastases of BC and CRC. IGF2BP2 can serve as a clinical inhibitory target for LC, AML, and CRC. IGF2BP3 can serve as a clinical inhibitory target for CRC and GC. YTHDC2 can serve as a clinical inhibitory target for LC. (By Figdraw.).

Taken together, the m^6^A methyltransferase METTL3 plays a critical role in regulating tumor angiogenesis in various cancers, including GC, CRC, liver cancer, BCa, and head and neck squamous cell carcinoma. This regulation significantly impacts tumor initiation, progression, invasion, and prognosis. Targeting METTL3 as a novel therapeutic and prognostic marker and developing highly specific inhibitors hold great promise for improving patient prevention, treatment, and survival rates in future clinical applications for these cancers. Notably, in renal cell carcinoma, METTL14 and IGF2BP2 collaboratively regulate tumor angiogenesis and metastasis while also managing drug resistance in cancer cells during clinical treatment. Consequently, we hypothesize that the development of METTL14-targeted inhibitors could have a substantial impact on the clinical management of renal cell carcinoma, paving the way for new therapeutic approaches. IGF2BP3 influences tumor angiogenesis by modulating different targets in various digestive system cancers. For example, in GC, IGF2BP3 enhances tumor angiogenesis and cell migration by promoting HIF1A expression. In contrast, in CRC, IGF2BP3 inhibits VEGF expression, thereby reducing tumor angiogenesis. Thus, IGF2BP3 can be targeted in gastric cancer and CRC cells to control tumor growth by inhibiting angiogenesis, achieving clinical treatment objectives. Notably, YTHDF3 promotes tumor angiogenesis in BC by facilitating the translation of m^6^A-enriched transcripts such as ST6GALNAC5, GJA1, and EGFR. Therefore, YTHDF3 has emerged as a potential therapeutic target for controlling the development and metastasis of BC (**Figure 3**).

## 5 Conclusions and Prospective

M^6^A modification is pivotal in regulating gene expression and extensively influences various cellular functions and disease processes. By modulating posttranscriptional modifications and RNA metabolism, m^6^A ultimately governs physiological and pathological processes within the body. Additionally, m^6^A interacts with histone methylation and acetylation, collaboratively regulating gene transcriptional activity and contributing to heterochromatin formation, thereby affecting chromatin structure and function to regulate gene expression. During tumorigenesis, m^6^A modification facilitates angiogenesis by regulating the expression of genes associated with tumor vascularization, thus supplying essential nutrients and oxygen to the tumor and accelerating its growth and metastasis. Recent studies suggest that ncRNAs interact with m^6^A to jointly influence processes such as tumor cell proliferation, migration, invasion, and angiogenesis. Targeting these genes and pathways presents an opportunity for developing m^6^A inhibitors for various cancers, potentially enabling personalized therapies and enhancing clinical outcomes. This discovery broadens the potential for targeting m^6^A as a novel therapeutic target in cancer and provides substantial evidence for the development of clinical anticancer drugs.

Despite the rapid progress in m^6^A research, several challenges persist. Researchers are working to determine the overall abundance of m^6^A in specific diseases, which will aid in disease diagnosis and prognosis evaluation and reveal the underlying pathological mechanisms involved. This information is vital for developing novel therapeutic strategies and advancing personalized medicine. Current studies have quantified the total m^6^A abundance in diseases such as abdominal aortic aneurysm (211), diabetic cataracts (212), NSCLC (213), HNSCC (214), and age-related cataracts (215), with the aim of precisely understanding the role of m^6^A in these conditions. However, there are currently no reports on the quantification of m^6^A abundance, which is specifically related to angiogenesis in particular tumors. Future research should focus on investigating whether dynamic changes in m^6^A abundance occur during tumor progression, whether the abundance and modification patterns of m^6^A vary at different growth stages and treatment phases, and whether these dynamic changes influence tumor angiogenesis and treatment responses.

Numerous studies have demonstrated that m^6^A methyltransferases and demethylases play critical regulatory roles in the progression of certain tumors. For instance, in AML, the m^6^A methyltransferase METTL3 is crucial for the myeloid differentiation of both normal and leukemic cells. Compared to healthy hematopoietic stem/progenitor cells, METTL3 expression is significantly elevated in AML cells. Inhibiting METTL3 in AML cells not only induces differentiation but also increases apoptosis and slows the progression of leukemia. This indicates that METTL3 is essential for maintaining the undifferentiated state of leukemic cells (114). Additionally, research by Zejuan Li et al. has shown that the m^6^A demethylase FTO plays a key oncogenic role in AML. FTO is highly expressed in AML subtypes carrying t(11q23)/MLL rearrangements, t (15, 17)/PML-RARA, FLT3-ITD, and/or NPM1 mutations. FTO enhances leukemogenesis and leukemia oncogene-mediated cell transformation by reducing m^6^A levels on mRNA transcripts of targets such as ASB2 and RARA, thereby regulating their expression and inhibiting all-trans retinoic acid (ATRA)-induced differentiation of AML cells. Notably, this study also found that FTO expression can be upregulated by certain oncogenic proteins, such as MLL fusion proteins, PML-RARA, FLT3-ITD, and NPM1 mutants, leading to abnormally high levels of FTO in these AML subtypes. This abnormal upregulation typically does not directly rely on m^6^A regulatory mechanisms (145). Taken together, a noteworthy phenomenon worth deeper exploration is that in the same tumor, m^6^A methyltransferases and demethylases may jointly regulate tumor development and progression, potentially exerting similar effects (either promoting or inhibiting). This phenomenon could be attributed to multiple factors. On the one hand, tumor development, cancer cell proliferation and migration, and angiogenesis often involve a vast and complex regulatory network, with multiple signaling pathways intertwined. Various components of the m^6^A machinery may participate in the modification of multiple genes and proteins, and while they may sometimes exhibit consistent functions, their specific target genes and regulatory pathways may differ, leading to this observed phenomenon. On the other hand, during this process, m^6^A writers and erasers are not solely governed by the m^6^A regulatory system. The human body is a complex organism with multi-level regulatory mechanisms, where the abnormal expression of certain oncogenes and proteins in the tumor microenvironment can influence the expression of writers and erasers, subsequently participating in regulation in an m^6^A-dependent manner. Therefore, the precise mechanisms by which m^6^A influences tumor progression and angiogenesis require more detailed and in-depth research to enable its application in clinical settings, ultimately becoming an effective therapeutic target to improve cancer patient survival rates.

In the future, developing targeted inhibitor treatment strategies for m^6^A in tumor angiogenesis will be crucial. In combination therapy strategies, m^6^A modification can affect tumor sensitivity to conventional therapies such as chemotherapy, radiotherapy, or immunotherapy, potentially enhancing therapeutic efficacy and reducing side effects. The levels of m^6^A modification and the expression of related factors could serve as biomarkers for tumor angiogenesis, aiding in the assessment of angiogenesis status, predicting disease progression, and guiding the formulation of individualized treatment plans. Further exploration of the specific mechanisms of m^6^A modification in different tumor types, particularly its impact on angiogenesis and the tumor microenvironment, as well as clinical trials of m^6^A-targeted drugs, is crucial. These trials should evaluate the efficacy, safety, and potential side effects of these therapies in cancer treatment. Personalized treatment strategies based on m^6^A modification hold promise for improving the precision and effectiveness of therapies, thereby enhancing the clinical outcomes of patients.

## Glossary

m^6^A: N6-methyladenosine
mRNA: messenger RNA
NcRNA: Non-coding RNA
MTC: m^6^A methyltransferase complex
FTO: Fat Mass and Obesity-associated
siRNA: Small interfering RNA
METTL3/5/7A/7B/14/16: Methyltransferase-like 3/5/7A/7B/14/16
TRMT112: tRNA MTase subunit 11–2
WTAP: the METTL3 adaptor protein wilms tumor 1-associated protein
RBM15: RNA-binding motif protein15
ZC3H13: Zinc finger CCCH domain-containing protein13
CBLL1: Cbl proto-oncogene like 1
ZCCHC4: zinc finger CCHC domain-containing protein 4
YTHDC1/2: YTH domain-containing 1/2
YTHDF1-3: YTH N6-methyladenosine RNA-binding protein 1-3
ALKBH5: AlkB homolog 5
VIRMA/KIAA1429: Vir-like m^6^A methyltransferase associated protein
IGF2BP2/3: Insulin-like growth factor 2 mRNA-binding protein 2/3
miRNA: microRNA
piRNA: PIWI-interacting RNA
circRNA: Circular RNA
LncRNA: long non-coding RNA
m^6^Am: 2’-O-dimethyladenosine
VEGF: Vascular endothelial growth factor
bFGF: basic fibroblast growth factor
VEGFR: vascular endothelial growth factor receptor
FGF: fibroblast growth factor
FGFR: fibroblast growth factor receptor
PI3K: phosphatidylinositol 3-kinase
PKB: protein kinase B
IRF5: interferon regulatory factor 5
PPP6C: protein phosphatase 6 catalytic subunit
TSCC: tongue squamous cell carcinoma
BATF2: basic leucine zipper ATF-like transcription factor 2
LUAD: lung adenocarcinoma
AML: acute myeloid leukemia
GC: gastric cancer
MTOR: mammalian target of rapamycin
BCa: bladder cancer
TEK: tie-2 receptor tyrosine kinase
VEGFA: vascular endothelial growth factor A
CRC: colorectal cancer
CCND1: CyclinD1
PDCD4: programmed cell death 4
MAPK: mitogen-activated protein kinases
GBM: glioblastoma
ERK: extracellular signal-regulated kinase
IL-8: Interleukin-8
HDGF: hepatoma-derived growth factor
HIF1a: hypoxia-inducible factor-1a
HCC: hepatocellular carcinoma
HUVECs: human umbilical vein endothelial cells
PC: pancreatic cancer
RARA: retinoic acid receptor alpha
MYC: myelocytomatosis oncogene
CEBPA: enhancer binding protein α
ICC: intrahepatic cholangiocarcinoma
OC: ovarian cancer
CC: cervical cancer
LC: lung cancer
TNS1: tensin 1
EC: endometrial cancer
TK1: thymidine kinase 1
VASH1: Vasohibin-1
BC: breast cancer
5-FU: 5-fluorouracil
KO: knockout
R-2HG: R-2-Hydroxyglutarate
CLIC4: recombinant chloride intracellular channel protein 4
NSCLC: non-small cell lung cancer
TRPM7: transient receptor potential melastatin 7
RCC: renal cell carcinoma
cRCC: clear cell kidney cancer
PEBP1: recombinant phosphatidylethanolamine binding protein 1
PLAU: plasminogen activator urokinase
EMT: epithelial-mesenchymal transition
VM: vasculogenic mimicry
ETV5: ETS variant transcription factor 5
TRAF1: Tumor Necrosis Factor Receptor (TNFR)-associated factor 1
GJA1: gap junction 1
IARC: the International Agency for Research on Cancer
